# Plasma amyloid beta levels are associated with cerebral amyloid and tau deposition

**DOI:** 10.1016/j.dadm.2019.05.007

**Published:** 2019-07-26

**Authors:** Shannon L. Risacher, Noelia Fandos, Judith Romero, Ian Sherriff, Pedro Pesini, Andrew J. Saykin, Liana G. Apostolova

**Affiliations:** a Department of Radiology and Imaging Sciences, Center for Neuroimaging, Indiana University School of Medicine, Indianapolis, IN, USA; b Indiana Alzheimer Disease Center, Indiana University School of Medicine, Indianapolis, IN, USA; c Araclon Biotech S.L., Zaragoza, Spain; d Department of Neurology, Indiana University School of Medicine, Indianapolis, IN, USA

**Keywords:** Blood biomarkers, Amyloid positron emission tomography (PET), Tau positron emission tomography (PET), Alzheimer's disease (AD), mild cognitive impairment (MCI)

## Abstract

**Introduction:**

We investigated the relationship of plasma amyloid beta (Aβ) with cerebral deposition of Aβ and tau on positron emission tomography (PET).

**Methods:**

Forty-four participants (18 cognitively normal older adults [CN], 10 mild cognitive impairment, 16 Alzheimer's disease [AD]) underwent amyloid PET and a blood draw. Free and total plasma Aβ40 and Aβ42 were assessed using a validated assay. Thirty-seven participants (17 CN, 8 mild cognitive impairment, 12 AD) also underwent a [^18^F]flortaucipir scan. Scans were preprocessed by standard techniques, and mean global and regional amyloid and tau values were extracted. Free Aβ42/Aβ40 (Aβ F42:F40) and total Aβ42/Aβ40 (Aβ T42:T40) were evaluated for differences by diagnosis and relation to PET Aβ positivity. Relationships between these measures and cerebral Aβ and tau on both regional and voxel-wise basis were also evaluated.

**Results:**

Lower Aβ T42:T40 was associated with diagnosis and PET Aβ positivity. Lower plasma Aβ T42:T40 ratios predicted cerebral Aβ positivity, both across the full sample and in CN only. Finally, lower plasma Aβ T42:T40 ratios were associated with increased cortical Aβ and tau in AD-related regions on both regional and voxel-wise analyses.

**Discussion:**

Plasma Aβ measures may be useful biomarkers for predicting cerebral Aβ and tau. Additional studies in larger samples are warranted.

## 1 Introduction

Alzheimer's disease (AD) is the most common neurodegenerative disease associated with aging. AD affects 5.7 million individuals in the United States, a number that is expected to rise to nearly 14 million by 2050 [1]. Early detection is increasingly considered critical, in that interventions designed to slow or stop disease progression during early stages are likely to be most effective. To identify individuals at risk for progression to AD, numerous biomarkers are being investigated, including neuroimaging measures, sensory measures, digital biomarkers, blood levels of target proteins, and many others.

Recent advances in blood-based assays have suggested that levels of amyloid beta (Aβ) can be precisely measured and are associated with levels of Aβ in the brain, making them good potential biomarkers for AD-risk screening and early detection. Specifically, previous studies have suggested that plasma levels of Aβ, tau, and other target proteins, such as neurofilament light, amyloid precursor protein, and others, are altered in patients with AD and those in the prodromal stage of AD, mild cognitive impairment (MCI). A number of studies have suggested that plasma Aβ42 and Aβ40 measures, as well as the ratio of Aβ42/Aβ40 are reduced in patients with AD and MCI and that these plasma biomarkers can predict the presence of AD and MCI and progression from normal to impaired cognition [2], [3], [4], [5], [6], [7], [8], [9], [10], [11], [12], [13], [14], [15], [16], [17], [18]. However, other studies have shown that higher plasma Aβ measures are associated with AD and conversion to AD [19], [20], [21], [22], [23], [24]. Still, others have found no significant relationship [25], [26]. Reduced plasma Aβ has also been linked to poorer cognition [6], [7], [19], [21], [27], [28], [29], [30]. In addition, some studies have suggested that plasma levels of Aβ42 and Aβ40 are associated with the levels of cerebral Aβ on positron emission tomography (PET), cerebrospinal fluid levels of Aβ and tau, and AD-like brain atrophy [4], [7], [8], [11], [16], [17], [24], [31], [32], [33], [34], [35], [36], [37], [38], while others have not seen such an association [25], [39], [40]. These conflicting results may be due to the type of plasma markers measured and the methodologies used to extract these values. For example, owing to its hydrophobicity, Aβ peptides interact with many proteins of the plasma matrix such as albumin, α2-macroglobulin, or lipoproteins among others [41], [42]. This could cause epitope masking, hindering the recognition of up to 50% of these amyloid peptides in the immunoassays [43]. This matrix effect could affect the reliability of Aβ peptide quantifications in an individual. To deal with this, we decided to measure both the free and total amount of these peptides in plasma in the present study. Another factor that may influence the relationship between plasma Aβ analyte levels and other outcomes is the role of genetic variation, particularly apolipoprotein E (*APOE*) [44]. Finally, a recent study also showed a significant association of the tau/Aβ42 ratio with tau PET in a largely cognitively normal (CN) Korean sample [37].

The goal of the present study was to investigate a measure of plasma Aβ42 and Aβ40 in a cohort of participants who are CN or are diagnosed with MCI or AD. Our initial goal is to replicate previous studies showing that plasma Aβ measures are linked to the presence of cerebral Aβ on PET. Furthermore, we extend these analyses to also investigate whether plasma Aβ42 and Aβ40 are associated with cerebral tau deposition on PET. The overall purpose of this study is to establish whether this plasma Aβ measure represents a promising biomarker for potential screening and early diagnosis of those at risk for AD.

## 2 Methods

### 2.1 Participants

Forty-four participants (18 CN, 10 MCI, 16 AD) from the Indiana Memory and Aging Study (IMAS) at the Indiana Alzheimer Disease Center were included in this study. All participants underwent an amyloid PET scan with either [^18^F]florbetapir or [^18^F]florbetaben, cognitive and clinical assessment, and a blood sample. Thirty-seven participants also were studied with tau PET using [^18^F]flortaucipir. Diagnoses were made by clinician consensus using standard criteria. Briefly, participants with MCI had a significant complaint about their cognition from themselves and/or an informant or clinician, as well as a significant deficit (>1.5 standard deviation below normal) in either memory or another cognitive domain, but with no significant decline in daily functioning. Patients with AD showed significant impairment on cognitive measures and a decline in daily functioning and met criteria for an AD diagnosis according to the updated National Institute of Neurological and Communicative Disorders and Stroke and the Alzheimer's Disease and Related Disorders Association criteria [45].

All procedures were approved by the Indiana University School of Medicine Institutional Review Board, and informed consent was obtained according to the Declaration of Helsinki and the Belmont Report.

### 2.2 Plasma Aβ assay

The Aβ assays to evaluate Aβ40 and Aβ42 (ABtest40 and ABtest42) were developed by Araclon Biotech Ltd (Zaragoza, Spain). These are two validated colorimetric tests based on a sandwich enzyme-linked immunosorbent assay technique, as previously described [43]. Aβ40 and Aβ42 are measured separately first in an undiluted plasma sample, which allows the detection of the amount of Aβ which is readily available for immunoassay detection (free in plasma, FP) and then in another aliquot of the same sample, diluted 1:3 in a proprietary buffer specifically formulated to break the interaction of the Aβ peptides with other plasma components (total in plasma fraction, TP). All the samples were analyzed in duplicates in a single assay to avoid interassay variability. In addition, all samples were processed blinded to the diagnostic group or amyloid status. We analyzed two ratios, free plasma Aβ42 to free Aβ40 (Aβ F42:F40) and total plasma Aβ42 to total Aβ40 (Aβ T42:T40), as they had been previously shown to correlate with amyloid positivity [5], [13], [16], [36].

### 2.3 Amyloid PET

[^18^F]Florbetapir (AmyVid; Eli Lilly and Co.) or [^18^F]florbetaben (Neuraceq; Piramal Ltd.) were acquired on all participants. Briefly, [^18^F]florbetapir scans were initiated by an intravenous injection of approximately 10 mCi of [^18^F]florbetapir. After a 50-minute uptake period, participants were imaged on a Siemens mCT for 20 minutes (50–70 minutes) using continuous list mode data acquisition. [^18^F]Florbetaben scans involved the intravenous administration of approximately 8 mCi of [^18^F]florbetaben. After a 90-minute uptake period, PET data were acquired for 20 minutes (90–110 minutes) using continuous list mode acquisition on a Siemens mCT. A computed tomography scan was acquired for both scans for scatter and attenuation correction. List mode data were subsequently rebinned into four 5-minute frames for both types of amyloid PET scans and reconstructed using parameters from the Alzheimer's Disease Neuroimaging Initiative protocol (http://adni.loni.usc.edu), with corrections for scatter and random coincidence events, attenuation, and radionuclide decay. The four 5-minute frames for each type of amyloid PET scan were spatially aligned to each subject's T1-weighted structural magnetic resonance imaging, motion corrected, and normalized to Montreal Neurologic Institute space, using Statistical Parametric Mapping 8 (SPM8). For [^18^F]florbetapir, the frames were averaged to create a 50-70 minute static image, while for [^18^F]florbetaben, the frames were averaged to create a 90–110 minute static image. Finally, static images were intensity normalized to the whole cerebellum to create standardized uptake value ratio (SUVR) images and smoothed with an 8-mm full-width half maximum Gaussian kernel. The whole cerebellum region of interest (ROI) was taken from the Centiloid project (http://www.gaain.org/centiloid-project/; [46]). [^18^F]Florbetapir and [^18^F]florbetaben scans were then processed with the Centiloid algorithm at a voxel-wise level as previously defined by the Centiloid project (http://www.gaain.org/centiloid-project/; [46]). Refer to the study by Risacher et al. [47] for more information. Regional [^18^F]florbetapir and [^18^F]florbetaben data (Centiloid units [CL]) were extracted from target ROIs, including the global cortex, lateral parietal lobe, and precuneus, generated using FreeSurfer version 5.1 (average of segmentations and parcellations from 30 CN older adult individuals from Alzheimer's Disease Neuroimaging Initiative-2) and extracted using MarsBaR [48]. A CL value of ≥10 was considered as amyloid positive [46], [49], [50].

### 2.4 [^18^F]Flortaucipir PET

The [^18^F]flortaucipir PET was initiated by intravenous injection of approximately 10 mCi of [^18^F]flortaucipir. After a 75-minute uptake, participants are imaged for 30 minutes by continuous list mode data acquisition on a Siemens mCT, which is subsequently rebinned into six 5-minute frames. Scans were again reconstructed using a standard scanner software program (Siemens, Knoxville, TN) and according to the Alzheimer's Disease Neuroimaging Initiative protocol (http://adni.loni.usc.edu). Using SPM8, the middle four 5-minute frames (80–100 minutes) were motion corrected, normalized to Montreal Neurologic Institute space using the subject-specific T1-weighted structural magnetic resonance imaging, averaged to create an 80–100 minute static image, intensity normalized to the cerebellar crus to create SUVR images, and smoothed with an 8-mm full-width half-maximum Gaussian kernel.

ROIs for target regions were generated from FreeSurfer v5.1 as described above. Specifically, bilateral mean SUVR values were extracted using MarsBaR [48] from the medial temporal lobe (average of fusiform gyri, parahippocampal gyri, and entorhinal cortex), lateral temporal lobe (average of inferior temporal gyri, middle temporal gyri, superior temporal gyri, banks of the superior temporal sulcus, and transverse temporal pole), and inferior parietal lobe.

### 2.5 Statistical analyses

Demographics and cognitive performance were compared among diagnostic groups using a one-way analysis of covariance (ANCOVA), covaried for age, sex, and years of education as appropriate. A chi-square test was used to evaluate differences by diagnostic group in noncontinuous variables (sex, ethnicity/race, *APOE* ε4 carrier status). The plasma Aβ measures of Aβ F42:F40 and Aβ T42:T40 were compared among diagnostic groups, using an ANCOVA model, covaried for age and sex. In addition, an ANCOVA was used to evaluate the effect of Aβ positivity (CL ≥ 10) on Aβ F42:F40 and Aβ T42:T40, covaried for age, sex, and diagnosis. Finally, an ANCOVA model was used to compare Aβ-positive MCI and AD with Aβ-negative CNs, covaried for age and sex, as these groups had a sufficient size for analysis. Bonferroni correction was applied to correct for multiple comparisons in all AN(C)OVA analyses. Logistic regression and receiver operating characteristic (ROC) curve analyses were used to predict amyloid positivity by plasma Aβ measures of Aβ F42:F40 and Aβ T42:T40 in the full sample and in CN participants only. Age and sex were tested as covariates in the logistic regression models but were nonsignificant. Finally, Pearson correlation models were used to evaluate the relationships between plasma Aβ measures of Aβ F42:F40 and Aβ T42:T40 and global and lateral parietal cerebral Aβ deposition, as these measures were all normally distributed. Amyloid in the precuneus and all tau regions did not show a normal distribution, and thus, they were converted to rank scores and their relationship with plasma Aβ measures of Aβ F42:F40 and Aβ T42:T40 were evaluated using Spearman models. Age and sex were not significantly associated with either the plasma amyloid or the regional cerebral amyloid and tau measures and, thus, were not included in the correlation analyses. All analyses were performed in Statistical Package for Social Sciences (SPSS) version 25 (https://www.ibm.com/products/spss-statistics).

Voxel-wise analyses were also performed to evaluate the linear relationship between plasma Aβ measures of Aβ F42:F40 and Aβ T42:T40 and cerebral Aβ and tau on a voxel-wise level. Multiple linear regression models were calculated using SPM8. All results were masked for gray plus white matter, and results are considered significant at a cluster-wise threshold of *P* < .05 (family-wise error [FWE] correction for multiple comparisons), which corresponds to a voxel-wise *P* < .001 (uncorrected) and minimum cluster size (k) = 550 voxels for amyloid and k = 800 voxels for tau.

## 3 Results

### 3.1 Demographics and cognitive performance

No differences were observed across diagnostic groups in age, sex, years of education, race/ethnicity, or *APOE* ε4 carrier status (Table 1). Expected differences in cognitive performance were observed, with patients with AD showing notable deficits compared with CN participants across cognitive domains. Patients with MCI showed deficits compared with CN participants on episodic memory, semantic fluency, and executive function/attention tasks (Table 1). Finally, significant differences in Aβ positivity were observed across diagnostic groups (Table 1; *P* < .001).

**Table 1:** Cohort characteristics

Characteristics	CN (n = 18)	MCI (n = 10)	AD (n = 16)	*P* value	Pairwise comparisons^a^
Age	67.7 (7.5)	69.0 (11.6)	65.9 (9.9)	ns	n/a
Sex (M, F)^b^	6, 12	5, 5	7, 9	ns	n/a
Education (years)	17.1 (2.5)	16.8 (3.3)	16.0 (2.5)	ns	n/a
Race/ethnicity (% non-Hispanic Caucasian)	77.8%	80.0%	75.0%	ns	n/a
*APOE* ε4 (% positive)^b^^c^	52.9%	77.8%	69.2%	ns	n/a
Aβ positivity (% positive)^b^	27.8%	80.0%	93.8%	<.001	MCI, AD > CN
CDR-sum of boxes	0.3 (0.6)	2.1 (1.4)	7.1 (4.6)	<.001	AD > MCI, CN
MoCA total score^d^^,^^e^	25.6 (2.4)	21.2 (3.7)	11.3 (6.8)	<.001	CN, MCI > AD
Digit span forward^d^^,^^f^	8.1 (1.7)	7.2 (1.5)	5.3 (2.8)	.004	CN > AD
Digit span backward^d^^,^^f^	6.9 (1.7)	5.9 (2.3)	3.9 (2.9)	.003	CN > AD
Digit symbol substitution^d^^,^^g^	53.3 (8.7)	37.1 (13.4)	28.6 (11.4)	<.001	CN > MCI, AD
Trail making part A (sec)^d^^,^^h^	32.3 (11.9)	37.9 (14.8)	43.1 (10.9)	ns	n/a
Trail making part B (sec)^d^^,^^h^	80.6 (21.8)	142.1 (17.5)	204.1 (21.6)	<.001	CN > MCI, AD
Animal fluency^d^^,^^i^	25.2 (5.6)	17.0 (5.4)	8.7 (6.0)	<.001	CN > MCI > AD
Vegetable fluency^d^^,^^j^	17.1 (5.2)	11.2 (4.5)	4.7 (4.1)	<.001	CN > MCI > AD
Letter fluency (F and L)^d^^,^^k^	30.2 (6.9)	26.8 (14.2)	20.0 (12.3)	ns	n/a
MINT total score^d^^,^^l^	30.0 (2.9)	28.1 (3.1)	23.7 (8.6)	.018	CN > AD
RAVLT immediate recall^d^^,^^m^	45.6 (6.0)	29.6 (9.5)	16.9 (9.1)	<.001	CN > MCI > AD
RAVLT delayed recall^d^^,^^m^	9.4 (2.2)	2.7 (3.7)	0.8 (1.6)	<.001	CN > MCI, AD
Craft stories immediate^d^^,^^n^	22.7 (6.7)	11.0 (8.2)	4.6 (3.2)	<.001	CN > MCI, AD
Craft stories delayed^d^^,^^n^	20.5 (7.0)	7.7 (7.3)	1.9 (3.0)	<.001	CN > MCI, AD
Benson figure copy^d^^,^^o^	16.1 (1.0)	15.2 (1.4)	11.9 (6.8)	.024	CN > AD
Benson figure recall^d^^,^^o^	12.7 (1.9)	5.5 (4.0)	2.6 (3.2)	<.001	CN > MCI, AD
Plasma Aβ FP42:FP40^p^	0.093 (0.017)	0.093 (0.020)	0.079 (0.017)	.047	None
Plasma Aβ TP42:TP40^p^	0.114 (0.019)	0.109 (0.015)	0.096 (0.016)	.016	CN > AD

NOTE. Values are shown as adjusted mean (standard deviation).

Abbreviations: AD, Alzheimer's disease; Aβ, amyloid beta; *APOE*, apolipoprotein E; CDR, Clinical Dementia Rating Scale; CN, cognitively normal; FP, free plasma; MCI, mild cognitive impairment; MINT, Multi-Lingual Naming Test; MoCA, Montreal Cognitive Assessment; n/a, not applicable; ns, not significant; RAVLT, Rey Auditory Verbal Learning Test; TP, total plasma.

a Bonferroni correction for multiple comparisons.

b Chi-square test.

c 5 participants missing data (1 CN, 1 MCI, 3 AD).

d Age, sex, and years of education included as covariates.

e 1 participant missing data (1 AD).

f 2 Participants missing data (2 AD).

g 8 Participants missing data (1 CN, 1 MCI, 6 AD).

h 9 Participants missing data (9 AD).

i 3 Participants missing data (1 CN, 2 AD).

j 4 Participants missing data (1 CN, 3 AD).

k 6 Participants missing data (1 MCI, 5 AD).

l 5 Participants missing data (5 AD).

m 9 Participants missing data (2 CN, 2 MCI, 5 AD).

n 4 Participants missing data (1 CN, 3 AD).

o 3 Participants missing data (3 AD).

p Age and sex includes as covariates.

### 3.2 Plasma Aβ F42:F40 and Aβ T42:T40 by diagnostic group and Aβ positivity

Regarding Aβ quantifications in plasma, intraplate coefficient of variability was 3.0% for ABtest40 and 4.6% for ABtest42. Interplate coefficients of variability were 4.9% and 6.1%, respectively. The dynamic range of the calibration curve ranged from 3.13 pg/mL to 200 pg/mL for ABtest40 and from 1.56 pg/mL to 100 pg/mL for ABtest42. Mean percentage calibration error was 2.2% for ABtest40 and 3.0% for ABtest42. The lower limit of quantification of the assays, empirically tested for precision and accuracy, are 7.6 pg/mL for ABtest40 and 3.6 pg/mL for ABtest42 [43]. All samples in our study were above these limits.

**Fig. 1:**
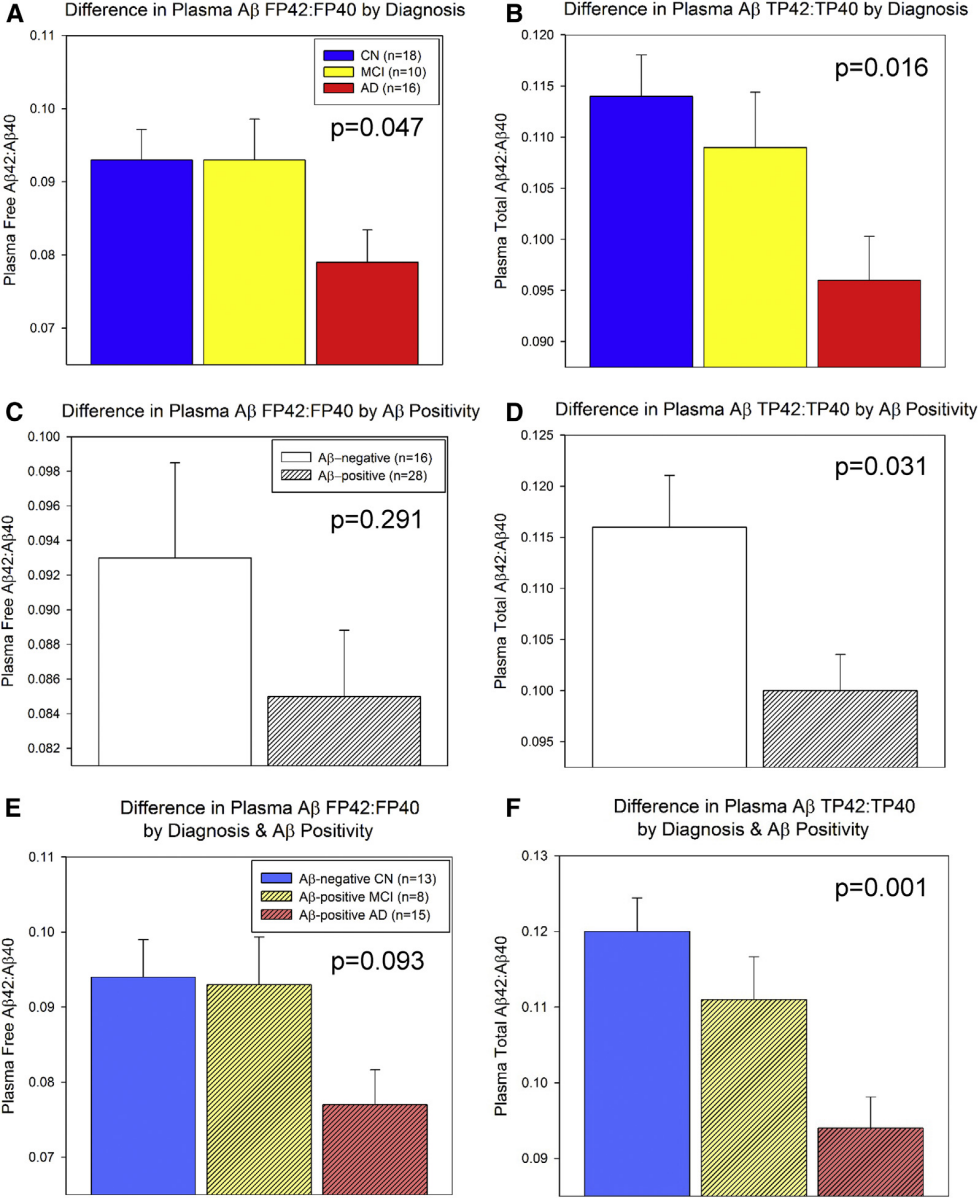
Differences in plasma Aβ measures by diagnosis and amyloid positivity. Plasma Aβ measures of free Aβ42-to-Aβ40 ratio (FP42:FP40; A) and total Aβ42 to Aβ40 ratio (TP42:TP40; B) were significantly different by diagnostic group, primarily driven by reduced values in patients with AD (both *P* < .05). When combined across groups, Aβ-positive individuals showed no significant difference from Aβ-negative individuals in FP42:FP40 (*P* > .05; C), but significantly lower TP42:TP40 (*P* = .031; D). Finally, Aβ-positive patients with AD had significantly lower FP42:FP40 (overall *P* value = .093; E) and TP42:TP40 (overall *P* value = .001; F) relative to Aβ-negative CNs (pairwise *P* ≤ .05 after Bonferroni correction). Results are shown as adjusted mean for each group with standard error as the error bars. Abbreviations: Aβ, amyloid beta; CN, cognitively normal; MCI, mild cognitive impairment; AD, Alzheimer's disease.

Plasma Aβ measures of Aβ F42:F40 and Aβ T42:T40 were significantly different among diagnostic groups (Table 1; Fig. 1A and B), driven primarily by reduced Aβ ratios in the AD group. When combined across groups, Aβ-positive individuals showed no significant difference from Aβ-negative individuals in FP42:FP40 (Fig. 1C), but significantly lower TP42:TP40 (Fig. 1D). In addition, there was a significant overall effect of group (Aβ-negative CN, Aβ-positive MCI, Aβ-positive AD) on TP42:TP40 (*P* = .001; Fig. 1F), but not on FP42:FP40 (Fig. 1E). Finally, Aβ-positive patients with AD had significantly lower FP42:FP40 (Fig. 1E) and TP42:TP40 (Fig. 1F) than Aβ-negative CN participants (pairwise *P* ≤ .05 after Bonferroni correction).

### 3.3 Prediction of PET cerebral Aβ positivity by plasma Aβ F42:F40 and Aβ T42:T40

**Fig. 2:**
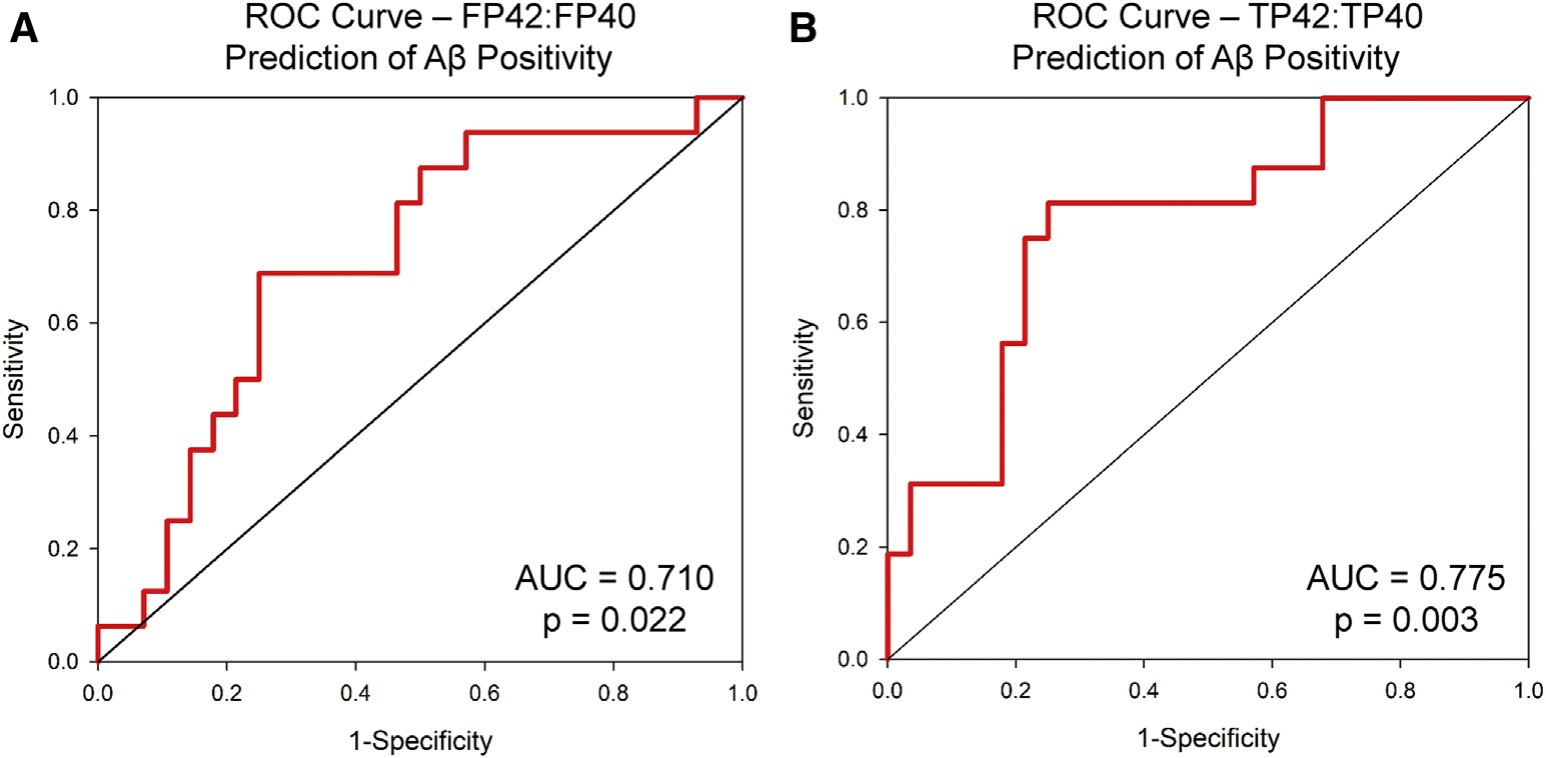
Predictive accuracy of plasma Aβ measures. Free plasma Aβ42-to-Aβ40 ratio (FP42:FP40) significantly predicted cerebral Aβ positivity across the full sample (*P* = .022, area under the curve (AUC) = 0.710; A). Total plasma Aβ42-to-Aβ40 ratio (TP42:TP40) also significantly predicted cerebral Aβ positivity across the full sample (*P* = .003, AUC = 0.775; B). Abbreviations: Aβ, amyloid beta; ROC, receiver operating characteristic.

**Supplementary Fig. 1:**
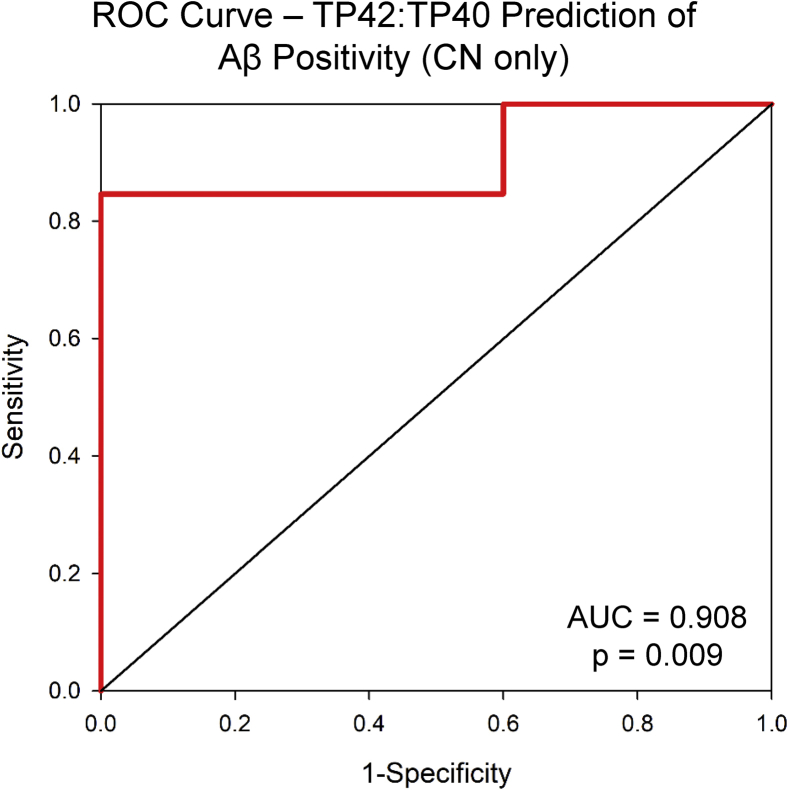
Predictive accuracy of plasma Aβ total Aβ42-to-Aβ40 ratio (TP42:TP40) for Aβ positivity in cognitively normal individuals. Plasma TP42:TP40 significantly predicts amyloid positivity in cognitively normal individuals (AUC = 0.908, *P* = .009). However, only five cognitively normal individuals (total n = 18) were amyloid positive in this cohort, which reduces the strength of this finding. Thus, this analysis should be repeated in a larger cohort of controls to validate this finding. Abbreviations: AUC, area under the curve; Aβ, amyloid beta; ROC, receiver operating characteristic; CN, cognitively normal.

In the full sample, both Aβ F42:F40 and Aβ T42:T40 predicted PET Aβ positivity using both a logistic regression model (Aβ F42:F40: *P* = .049, 68.2% accuracy; Aβ T42:T40: *P* = .005, 70.5% accuracy) and ROC curves (Aβ F42:F40: area under the curve [AUC] = 0.710, *P* = .022, Fig. 2A; Aβ T42:T40: AUC = 0.775, *P* = .003, Fig. 2B). Although only five CN participants were Aβ positive, Aβ T42:T40 predicted cerebral Aβ positivity in CNs with 100% accuracy in the logistic regression model and had an AUC of 0.908 in the ROC curve model (Supplementary Fig. 1; *P* = .009). However, this finding must be interpreted with caution given the small sample size, and additional studies in larger samples are warranted.

### 3.4 Associations between plasma Aβ F42:F40 and Aβ T42:T40 and regional cerebral Aβ and tau deposition on PET

**Fig. 3:**
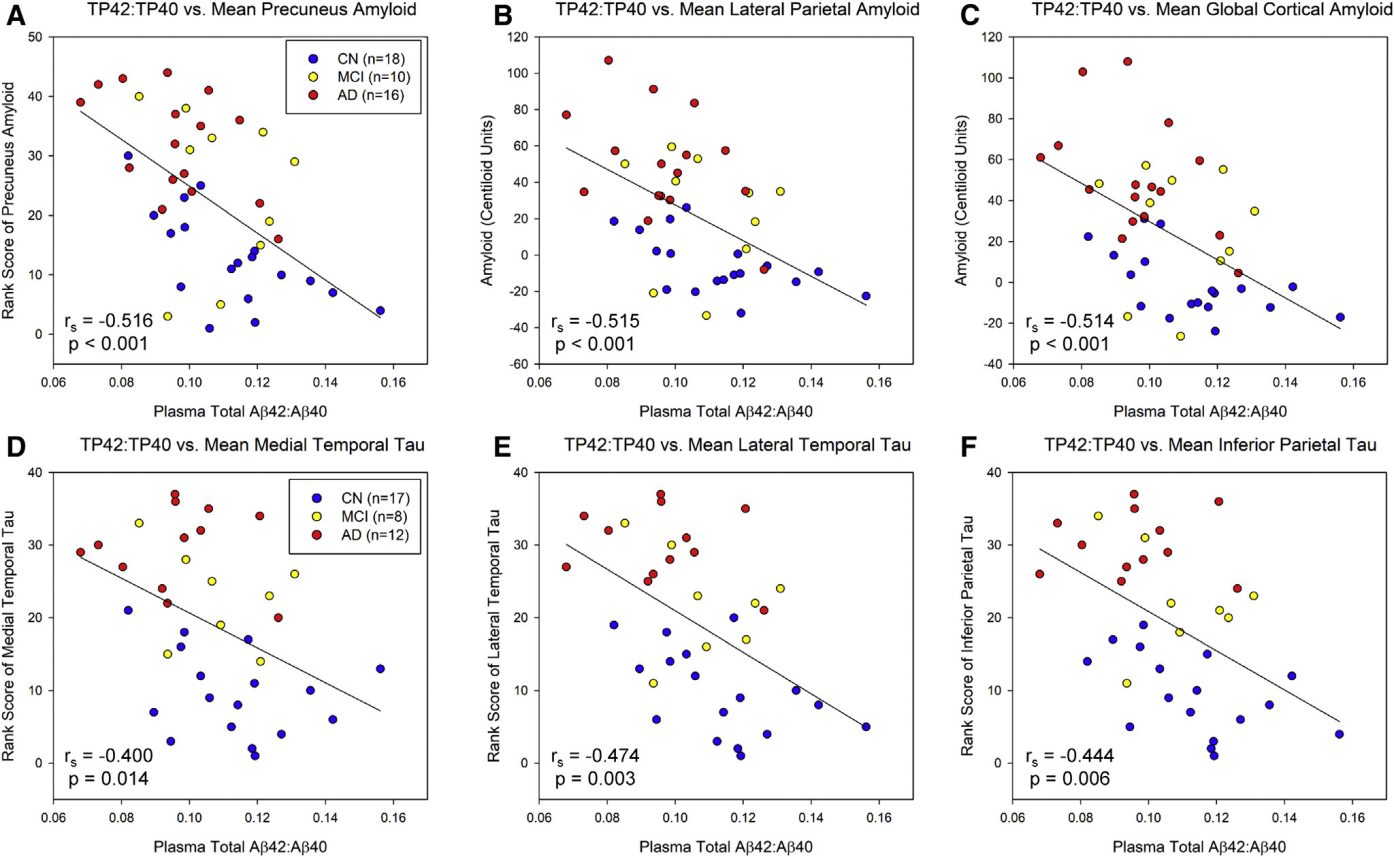
Relationship between plasma total Aβ42-to-Aβ40 ratio (TP42:TP40) and regional cerebral amyloid deposition and tau deposition on PET. Significant linear relationships between TP42:TP40 and cerebral amyloid deposition in the bilateral precuneus (rs = −0.516, *P* < .001; A), lateral parietal lobe (r = −0.515, *P* < .001; B), and global cortex (r = −0.514, *P* < .001; C) were observed. TP42:TP40 was significantly associated with cerebral tau deposition (rank scores of [18F]flortaucipir SUVR) in the bilateral medial temporal lobe (rs = −0.400, *P* = .014; D), lateral temporal lobe (rs = −0.474, *P* = .003; E), and inferior parietal lobe (rs = −0.444, *P* = .006; F). The analyses of lateral parietal and global cortical amyloid (B and C) are Pearson correlations, while the other analyses are Spearman correlation models (A, D-F). Abbreviations: PET, positron emission tomography; Aβ, amyloid beta; SUVR, standardized uptake value ratio; CN, cognitively normal; MCI, mild cognitive impairment; AD, Alzheimer's disease.

**Supplementary Fig. 2:**
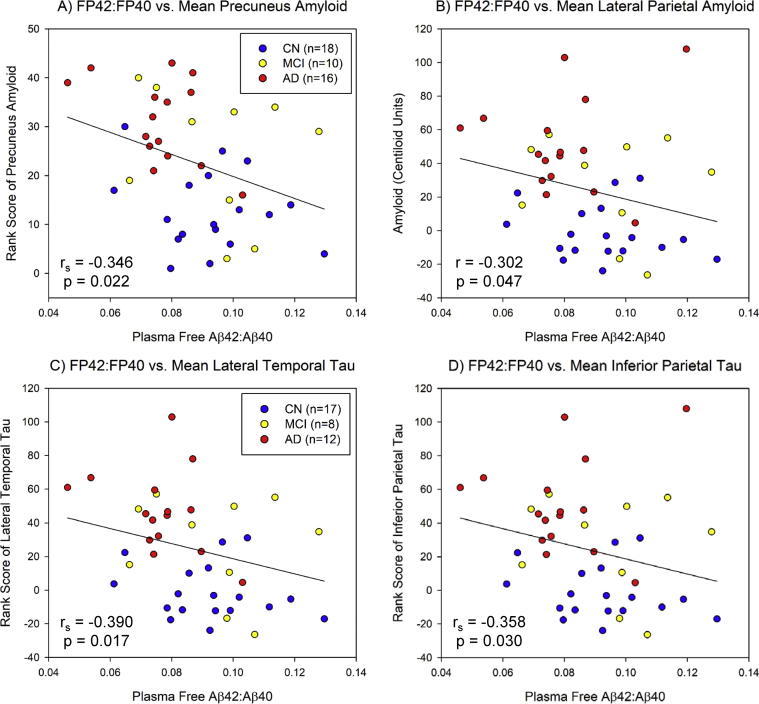
Relationship between plasma free Aβ42-to-Aβ40 ratio (FP42:FP40) and regional cerebral amyloid deposition and tau deposition on PET. Significant relationships between FP42:FP40 and cerebral amyloid deposition in the bilateral precuneus (r_s_ = −0.400, *P* = .022; A) and lateral parietal lobe (r = −0.302, *P* = .047; B) were observed across participants. In addition, FP42:FP40 was associated with tau deposition in the bilateral lateral temporal lobe (r_s_ = −0.390, *P* = .017; C) and inferior parietal lobe (r_s_ = −0.358, *P* = .030; D). Abbreviations: CN, cognitively normal; MCI, mild cognitive impairment; AD, Alzheimer's disease; Aβ, amyloid beta.

Significant associations between plasma Aβ T42:T40 and cerebral Aβ deposition were observed in the precuneus (Fig. 3A; r_s_ = −0.516, *P* < .001), lateral parietal lobe (Fig. 3B; r = −0.515, *P* < .001), and global cortex (Fig. 3C; r = −0.514, *P* < .001). In addition, significant associations between plasma Aβ F42:F40 and amyloid in the precuneus (Supplementary Fig. 2A; r_s_ = −0.346, *P* = .022) and lateral parietal lobe (Supplementary Fig. 2B; r_s_ = −0.302, *P* = .047) were observed.

Significant associations were also observed between plasma Aβ T42:T40 and cerebral tau deposition in the medial (Fig. 3D; r_s_ = −0.400, *P* = .014) and lateral (Fig. 3E; r_s_ = −0.474, *P* = .003), temporal, and inferior parietal lobes (Fig. 3F; r_s_ = −0.444, *P* = .006). In addition, significant associations between plasma Aβ F42:F40 and lateral temporal (Supplementary Fig. 2C; r_s_ = −0.390, *P* = .017) and inferior parietal lobe tau (Supplementary Fig. 2D; r_s_ = −0.358, *P* = .030). However, if amyloid load (global cortex CL) is included as a covariate in the association between plasma Aβ 42:40 (both total and free) and tau deposition, the correlations are no longer significant.

### 3.5 Voxel-wise associations between plasma Aβ F42:F40 and Aβ T42:T40 and cerebral Aβ and tau deposition

**Fig. 4:**
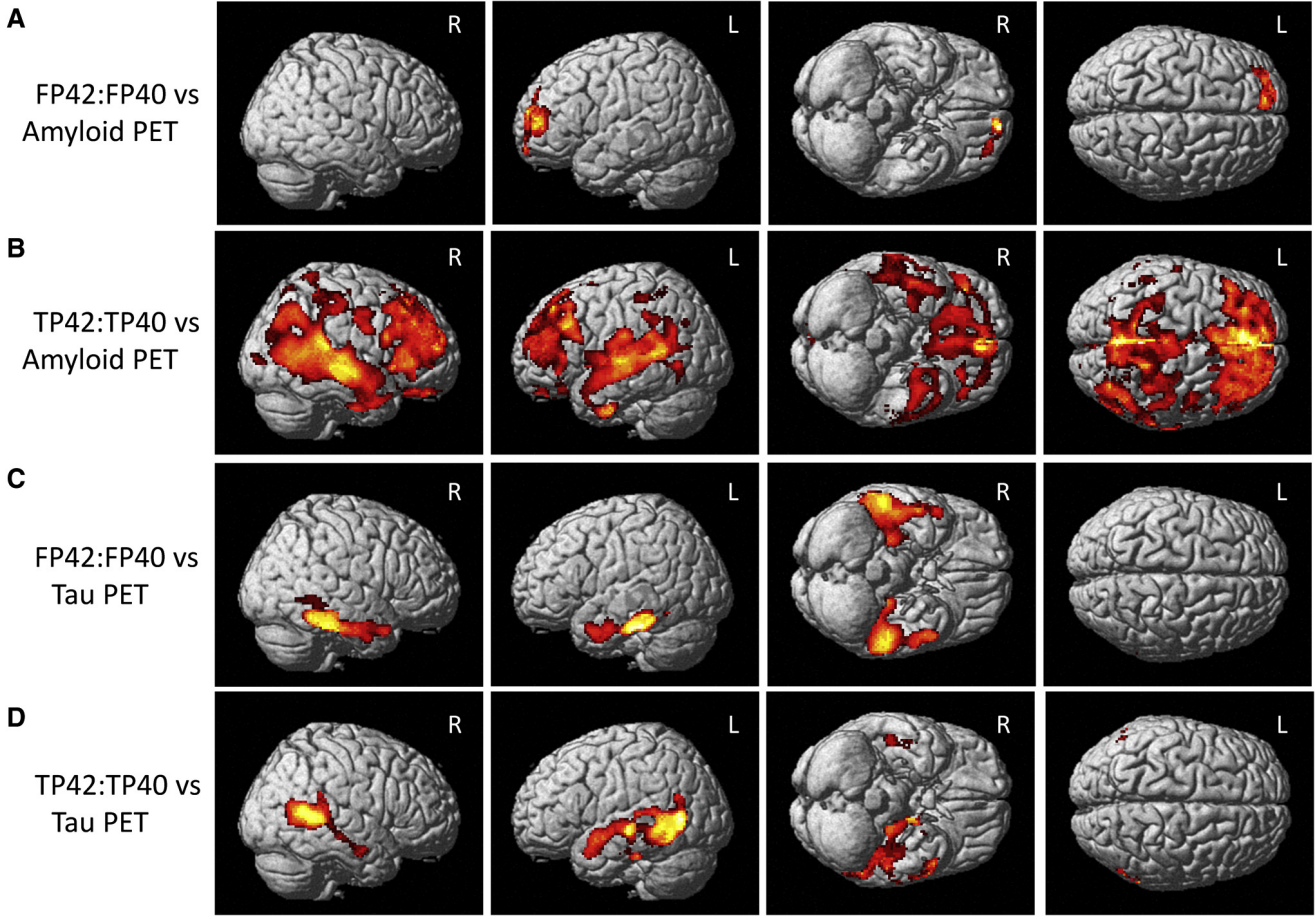
Voxel-wise relationship between plasma Aβ measures and cerebral amyloid and tau deposition on PET. A significant relationship between plasma free Aβ42-to-Aβ40 ratio (FP42:FP40) and cerebral amyloid deposition in the right lateral temporal lobe and bilateral frontal lobe was observed (A), while plasma total Aβ42-to-Aβ40 ratio (TP42:TP40) was associated with cerebral amyloid throughout the cortex (B). FP42:FP40 was also associated with cerebral tau in the bilateral medial and lateral temporal and right inferior parietal lobe (C). Finally, TP42:TP40 was associated with cerebral tau in the bilateral medial and lateral temporal and parietal lobes, as well as the bilateral posterior frontal lobe (D). All images are displayed at a cluster-wise corrected *P* < .05 and masked for gray and white matter. Abbreviations: PET, positron emission tomography; Aβ, amyloid beta.

Plasma Aβ F42:F40 was significantly associated with amyloid deposition in the left frontal lobe on voxel-wise analysis (Fig. 4A; cluster-wise *P* < .05 [FWE]). A larger area of amyloid deposition encompassing nearly the entire cortex was significantly associated with plasma Aβ T42:T40 (Fig. 4B; cluster-wise *P* < .05 [FWE]).

Cerebral tau deposition in the bilateral medial and lateral temporal lobes was significantly associated with plasma Aβ F42:F40 on voxel-wise analysis (Fig. 4C; cluster-wise *P* < .05 [FWE]). Plasma Aβ T42:T40 was significantly associated with tau deposition in the temporal and parietal lobes (Fig. 4D; cluster-wise *P* < .05 [FWE]).

## 4 Discussion

We observed significantly lower levels of plasma Aβ T42:T40 in patients with AD than in CN older adults, as has been previously reported [2], [3], [4], [5], [6], [7], [8], [9], [10], [11], [12], [13], [14], [15], [16], [17], [18]. In addition, a trend for lower plasma Aβ F42:F40 in patients with AD was also observed. This observed difference could be accounted for by cerebral Aβ positivity on PET within the diagnostic groups. Both plasma Aβ F42:F40 and T42:T40 predicted Aβ positivity across the full sample. Although interpretations are limited by sample size (n = 5 amyloid positive), only plasma Aβ T42:T40 predicted cerebral Aβ positivity in the CN group, suggesting perhaps that total levels may be more sensitive in early stages. Significant linear associations between plasma Aβ F42:F40 and Aβ T42:T40 and cerebral Aβ deposition were observed on both regional and voxel-wise analyses. These findings are similar to the previous report using this assay by Fandos et al. (2017). Finally, this study was one of the first to show significant associations between plasma Aβ F42:F40 and Aβ T42:T40 and cerebral tau deposition on both regional and voxel-wise analyses. These findings support recently reported findings showing that plasma t-tau, p-tau, and the ratios of t-tau/Aβ42 and p-tau/Aβ42 are associated with tau deposition on PET, as well as longitudinal changes in cerebral amyloid and neurodegeneration [37].

Plasma measures of amyloid, tau, and neurofilament light are increasingly showing a positive predictive value for AD-related neuropathology in patients with MCI and AD, as well as in preclinical AD. In the present analysis, we observed a strong relationship between plasma Aβ ratios of Aβ F42:F40 and Aβ T42:T40 and cerebral Aβ deposition, measured both as Aβ positivity and by actual Centiloid value. The strength of this association suggests that these measures of plasma Aβ may be good biomarkers for screening individuals for the presence of cerebral Aβ, even CN individuals. However, future studies are needed to fully investigate these markers in clinical populations and in larger and more diverse samples.

We also reported significant associations between plasma Aβ F42:F40 and Aβ T42:T40 and cerebral tau deposition in an AD-like pattern. However, including cerebral amyloid Centiloid values in a regression model predicting tau deposition with either plasma Aβ F42:F40 or Aβ T42:T40 alters the relationship such that only cerebral Aβ deposition significantly predicts tau. These findings suggest that cerebral amyloid is mediating the association between plasma Aβ F42:F40 and Aβ T42:T40 and tau. In other words, the plasma measure of Aβ is a proxy measure for brain amyloid deposition, and brain amyloid deposition is highly linked to cerebral tau deposition, thereby leading to an association between plasma Aβ and cerebral tau. However, future studies in larger samples could use mediation and moderation analyses to more fully ascertain the relationship between the plasma Aβ measures and cerebral amyloid and tau.

This study has a few notable limitations. First, the sample size is small. Future studies exploring larger and more diverse samples are warranted. Second, we did not consider other central and peripheral diseases (i.e., cerebrovascular disease, infection, liver and kidney function, etc.) that may alter the relationship between plasma Aβ and cortical amyloid and tau. Additional studies to investigate the impact of comorbidities on this relationship are crucial for ultimately establishing this assay as a clinical tool. In addition, the CN group in this study had a high prevalence of APOE ε4 positivity, suggesting that they represent a higher risk group. Thus, the findings in this study may not accurately generalize to the normal older adult population as a whole. Future epidemiologic studies in community-based samples are needed to explore plasma amyloid measures in the general older adult population. Finally, this is a cross-sectional study, and thus, we could not assess outcome data or whether the assay predicted future cognitive decline. Future studies will allow us to fully assess the outcome of these participants. In addition, future studies should investigate genetic associations and potential genetic modulators of plasma amyloid levels.

In sum, plasma Aβ measures were reduced in patients with MCI and AD, predicted cerebral Aβ positivity on PET, even in CN individuals, and were associated with cerebral amyloid and tau load. These preliminary findings suggest that these plasma measures of Aβ may be a potential screening tool for detecting AD-related neuropathology in at-risk individuals in clinical settings or pharmaceutical trials. As AD therapeutics are developed, these assays may be helpful in providing an initial determination of which individuals may benefit from follow-on cerebrospinal fluid and/or PET investigations before treatment.

**Research in context**

1. Systematic review: To investigate associations between plasma amyloid biomarkers and neuroimaging measures of cerebral amyloid and tau, we searched for combinations of “plasma,” “blood,” “amyloid,” “PET,” and “Alzheimer's.” We then combined the returned articles to generate a summary of the current literature evaluating blood-based biomarkers of AD in predicting diagnosis and abnormal neuroimaging and cerebrospinal fluid biomarkers.
2. Interpretation: Our results provide new evidence that plasma amyloid measures accurately reflect cerebral amyloid deposition and can predict the presence of amyloid in cognitively normal older adults with high accuracy. Furthermore, these results suggest a relationship of plasma amyloid with cerebral tau, which is mediated by cerebral amyloid.
3. Future directions: To confirm the current findings, additional analyses with larger samples would be beneficial. In addition, longitudinal follow-up studies with repeated plasma samples, neuroimaging, and cognitive testing would help determine whether the plasma amyloid measure can predict and monitor clinical decline over time.
